# Collective memory: between individual systems of consciousness and social systems

**DOI:** 10.3389/fpsyg.2023.1238272

**Published:** 2023-10-12

**Authors:** Jean-François Orianne, Francis Eustache

**Affiliations:** ^1^Center for Research and Sociological Interventions (CRIS), Social Science Research Institute (IRSS), Liège University, Liège, Belgium; ^2^Neuropsychology and Imaging of Human Memory (NIMH) Research Unit, GIP Cyceron, INSERM U1077, Caen University Hospital, PSL, EPHE, Caen University, Caen, France

**Keywords:** collective memory, shared memory, collaborative memory, social memory, autobiographical memory, individual memory, script, social role

## Abstract

Following a long period of neglect, research on different facets of collective memory is now developing apace in the human and social sciences, as well as at their interface with psychology and neuroscience. This resolutely multidisciplinary renewal of interest in memory sciences has given rise to a plethora of concepts with diverse meanings (e.g., social frameworks of memory, collective, shared, collaborative, social memory). The purpose of the present study was to provide a conceptual overview from a historical perspective, and above all to clarify concepts that are often used interchangeably, even though they refer to very different realities. Based on recent research in psychology and neuroscience, we use the concept of *collective memory* to refer to the operations of individual systems of consciousness. Collective memory is not the memory of a collective, but that of its individual members, either as members of social groups (*shared memory*) or as participants in social interactions (*collaborative memory*). Drawing on the contributions of contemporary sociology, we show that *social memory* is not collective memory, as it refers not to individual systems of consciousness, but to social systems. More specifically, it is the outcome of communication operations which, through redundancy and repetition, perform a continuous and selective re-imprinting of meaning that can be used for communication. Writing, printing and the new communication technologies constitute the three historical stages in the formation and development of an autonomous social memory, independent of living memories and social interactions. In the modern era, mass media fulfill an essential function of social memory, by sorting between forgetting and remembering on a planetary scale. When thinking about the articulation between collective memory and social memory, the concept of *structural coupling* allows us to identify two mechanisms by which individual systems of consciousness and social systems can interact and be mutually sensitized: schemas and scripts, and social roles. Transdisciplinary approach spearheads major methodological and conceptual advances and is particularly promising for clinical practice, as it should result in a better understanding of memory pathologies, including PTSD, but also cognitive disorders in cancer (chemobrain) or in neurodegenerative diseases.

## 1. Introduction

The concept of *collective memory* has been the subject of numerous theoretical debates and a wide range of empirical studies. Its suggestive power has inspired pioneering work on memory in the social sciences and, more recently, a *social turn* in the fields of psychology and cognitive neuroscience. This social turn has led to the renewal of the conceptual apparatus in the memory studies, with multiple concrete repercussions in fields as diverse as neuropsychology, neuroimaging and psychopathology. This renewal of memory sciences, characterized by multidisciplinary approaches, has given rise to a plethora of concepts with multiple meanings, often used interchangeably even though they designate processes that operate at very different levels.

In the present article, we seek to provide a conceptual overview, by reviewing the *wide-angle* literature from a historical perspective. We also make several conceptual clarifications we feel are essential in such a teeming field of research conducted “between chaos and diversity” (Olick, 2009). This includes making distinctions between collective memory and social memory frameworks, and between shared memory and collaborative memory.

We trace the concept back to the foundational writings of Durkheim and Halbwachs. As neither of these authors could adduce sufficient theoretical and empirical evidence in support of their original intuitions, they used metaphors (or reifications) to convince their readers and distill their innovative and stimulating ideas. Ambiguity, imprecision, confusion and profusion are clearly the price to pay for these theoretical innovations, which have left a lasting mark on memory research in the humanities and social sciences. Some authors have even gone so far as to suggest that the concept of collective memory should be abandoned once and for all, arguing that it is nothing more than a passing fad based on mystification (Gedi and Elam, 1996).

We certainly do not suggest abandoning the concept. Rather, our aim is to clarify and articulate the concepts of collective memory and social memory, reserving the concept of *social memory* for the communication operations of social systems, and the concept of *collective memory* for the operations of individual systems of consciousness. This may seem rather counterintuitive at first, but it allows us to escape from the metaphor of collective memory as the memory of a collective, given that it is actually the memory of individuals as members of a collective. In order to achieve this theoretical objective, we draw on recent work in psychology and neuroscience as well as on the contributions of contemporary sociology.

As we point out, one essential contribution of psychology is to go beyond the metaphorical character of the concept of collective memory by substituting two operational concepts: that of shared memory, and that of collaborative memory. *Shared memory* refers to individuals as members of a group, whereas *collaborative memory* refers to individuals as participants in an interaction. As for contemporary sociology, it offers the concept of *social memory*. By no means metaphorical, the latter refers to the memory of a society or of social systems, which of course are composed solely of self-produced and self-referential communication operations, and absolutely not of individuals and their individual and collective memories. As we demonstrate, social memory is generated by the recursiveness of communication operations, the repetition of the same references, and the continuous and selective re-imprinting of the system’s own states and meanings for use in subsequent communication operations.

We end by examining the articulation or interaction between collective memory and social memory. We then identify two very specific mechanisms of structural coupling between individual systems of consciousness and social systems: schemas and scripts on the one hand, and social roles on the other hand.

## 2. The origins of the concept: from Durkheim to Halbwachs

Émile Durkheim, the founding father of French sociology, and his disciple Maurice Halbwachs laid the theoretical foundations for the study of memory in the human and social sciences (Hutton, 1988; Llobera, 1995; Misztal, 2003). The rather general idea of a social or collective memory or consciousness that goes beyond individuals had already been mooted in Antiquity (Russell, 2006). It returned in force, in the literature and the press, in the second half of the 18th century, with the advent of modern society, along with other similar notions (e.g., *public spirit* or *popular will*) that were also based on an analogy between society and personality (Vincent, 1916). However, it was only at the turn of the 20th century that these notions penetrated the scientific vocabulary, notably in the nascent field of sociology, which defined itself in France as a positive and critical science (Durkheim, 1950). It should be noted that during this time, psychologists, psychiatrists and neurologists were trying to study and model individual memory stricto *sensu* (i.e., totally decontextualized, including its social aspects). Hermann Ebbinghaus, who studied memory and forgetting based on lists of meaningless syllables, is the most emblematic of these figures (Ebbinghaus, 2010).

### 2.1. A memory of society?

In *The Elementary Forms of Religious Life*, originally published in 1912, Durkheim (1960) described the main ritual attitudes, and more particularly commemorative rites, at some length. For example, Durkheim described a ceremony performed by the Warramunga, an aboriginal people of northern Australia, as commemorating and representing the mythical history of the ancestor. Durkheim emphasized that the officiant was regarded not as the incarnation of the divinity, but as an actor playing a role, who participated in a process of communication whose function was to make the past available to the present and thus to ensure the continuity of traditions. Songs, dances and mimes were important adjuncts to communication, lending rhythm to the narration of the mythical story. Durkheim also highlighted the mythical places where the events in the ancestor’s history took place, as these were represented in drawings on the ground or body paintings, and served as a communication medium, acting as topographical memories.

Without explicitly naming it, Durkheim therefore laid the foundations for the concept of *social memory*, in other words, the ability of a social system (group, clan, community, etc.) to remember in an autonomous way (independently of what the individual members remember), and manufacture memories (symbolic and topographical) that resist the passing of generations and guarantee the clan, tribe, or group a certain moral and semantic continuity. This sociological innovation in the study of memory was part of a visionary movement guided by an original intuition according to which society is a *sui generis* (i.e., self-produced) reality. It was in *The Rules of Sociological Method* that Durkheim established the concept of *social fact* as a reality that was autonomous of psychic and organic processes (Durkheim, 1950, p. 5).

### 2.2. Collective memory or social memory?

The concept of *collective memory* was introduced by Halbwachs in the 1920s and 1930s. This French sociologist based his sociological study of memory on his original intuition that *one never remembers alone*: “it is in this sense that there would exist a collective memory and social frameworks of memory, and it is insofar as our individual thought places itself in these frameworks and participates in this memory that it would be capable of remembering” (Halbwachs, 1925, Foreword). In this excerpt, a distinction is made between collective memory and social frameworks of memory: *collective memory* is the memory in which individuals participate, while *social frameworks* are the cognitive and normative structures of various social systems (religious, family, school, professional institutions, etc.). It is worth noting Halbwachs’ reference to individual thought, and above all to the junction between collective and individual memory, and more particularly recollection (which has since come to be known as episodic memory). Individual memory is based on a memory that transcends the individual, and is even made possible by it. Halbwachs’ study of *collective memory among musicians* emphasized the importance of social frameworks of memory, without which the formation of musical memories (individual and collective) would be impossible (Box 1).

BOX 1 **The Musicians’ collective memory (Halbwachs, 1939).**

Halbwachs’ thesis was that social systems provide individuals with repertoires (what he called *schemas external to the individual*) that enable them to continuously attribute meaning to noise, retain and organize sound stimuli, and transform them into personal musical memories that can be shared. Solfeggio (and more broadly musical knowledge) as an institution organizes meaning and sensitizes the organism to meanings through conventions, values that guide action, and communication. This (sub)system of values allows for the modulation of selection activities: “In any case: isolate the musician, deprive him of all these means of translation and fixation of sounds that musical writing represents: it will be very difficult and almost impossible for him to fix in his memory such a large number of memories” (Halbwachs, 1939, p. 144). There is an obvious link here to semantic memory, as well as to the schema theory developed by the British psychologist Frederic Bartlett (1932).

To use the terminology coined by Halbwachs and his contemporaries, the link between organism and personality is mediated by the social system through (musical) instruments, roles (pianist, conductor, amateur, etc.), and norms and values (musical theory, conventions of writing and interpretation, etc.). This *institution of musical meaning* modulates the activity of the organic systems (auditory, muscular, articular, respiratory, etc.) and gives the psychic system the opportunity to consciously experience (and remember) musical moments. Beyond the links between the organic system and the psychic system, which are difficult to conceptualize today, Halbwachs’ proposal underlines the relationship between semantic memory and episodic memory, with the latter relying on the former. Semantic memory, which is both individually and socially founded, serves as a junction, in terms of both access to and formation of knowledge.

The social frameworks of memory must not be confused with the (individual and collective) memory of musicians. If people remember more or less the same facts when they share a common experience, it is because a social system that is external to them gives them the opportunity to do so (Halbwachs, 1947). Here, this is the institution of musical meaning: a social memory that is very specific and largely independent of what the musicians remember or how they organize or refresh their memories. It is interesting to note that Halbwachs (1950) himself favored the notion of social memory over that of collective memory, despite what posterity has retained (Sabourin, 1997). Halbwachs’ distinction between collective memory and social memory may have lacked precision, but it was no less essential on a historical and analytical level. The way was now open for authors to explore the distinctions between internal and external memory, and between autobiographical and historical memory in greater depth, although these concepts remained highly unstable.

### 2.3. Collective memory after Halbwachs

This lack of precision seems to have been the hallmark of research in the human and social sciences. Although Halbwachs’ definitions of collective memory quickly fell into oblivion (Apfelbaum, 2010), it was nonetheless a foundational concept in memory studies, being to memory studies what the apple was to Newtonian physics!

Since the 1980s, the term *collective memory* has been used by social scientists to refer to phenomena as varied as memories and shared narratives (Knapp, 1989; Smith, 2004), commemorations (Schwartz, 1982, 1997; Ghoshal, 2013), myths and cultural scripts (Green, 2004), and culture (in the broadest sense). Serving more as a metaphor than as a clearly defined concept, it refers both to *collective representations* (worldviews, patterns or schemas, scripts, ideas, belief models) that organize the making of sense of individual memories (coding, semanticization), and to *collective practices* (social, cultural, community, etc.) that aim to externalize (inscribe, share, discuss, etc.) individual memories in an interindividual framework (Olick, 1999).

In the field of cognitive neuroscience, the infatuation with Halbwachs’ work has inspired a true *social turn* (Coman et al., 2009; Rajaram and Pereira-Pasarin, 2010; Saxena and Morris, 2016; Fischer and O'Mara, 2022). The concept of collective memory lies at the heart of the most heated debates (Laikhuram, 2022): Metaphor or reality? Are individuals the only ones who can remember? Do groups have a consciousness, a mind, a specific memory? Can we reasonably support the hypothesis of an *extended mind* that goes *beyond the individual* (Wilson, 2005)? According to Bachleitner (2022, p. 167), “in its collective form, memory thus is lifted out of the minds and brains of individuals and instead is socially generated in societal frameworks.” According to this *externalist* perspective, the concept of collective memory is similar to that of *joint memory*, understood as the property of a social group to actualize its own past transformations (Thierry et al., 1995). It can result from the coupling of individual memories (shared or interindividual memories) and/or from couplings with the social or cultural environment (social or cultural memories).

In the wake of this new enthusiasm for a rereading of Halbwachs’ work, some people questioned the actual added value of the concept of collective memory, and criticized its metaphorical use in what were very vague conceptual and theoretical frameworks. According to Kansteiner (2002), this burgeoning field of research had made virtually no theoretical progress, and had instead accumulated methodological issues linked to the metaphorical use of psychological and neurological vocabulary to explain collective memory as an *extension* of individual memory. Wass metaphor therefore just a catch-all category, with a high potential for scientific emulation around the idea of transdisciplinarity, when even within individual disciplines there was no real agreement on what the concept should cover (Confino, 1997)? Why multiply related notions (shared, common, discursive, collaborative memories, etc.) in the absence of any mapping and delimitation? For Olick and Robbins (1998), social memory studies were a “nonparadigmatic, transdisciplinary, and centerless” (p. 106) research enterprise that navigated in troubled waters “between chaos and diversity” (Olick, 2009). Gedi and Elam (1996) even called for the concept of collective memory to be abandoned altogether, arguing that it was simply a passing fad based on mystification.

Our proposal is not to abandon the concept, but to clarify and articulate the concepts of collective memory and social memory, reserving the concept of *social memory* for the communication operations of social systems, and the concept of *collective memory* for the operations of individual systems of consciousness. This may seem counterintuitive, but it allows us to extract ourselves from the metaphor of collective memory as the memory of a group, given that it is actually the memory of individuals as members of a collective. To justify this proposal, we will draw on recent research in psychology and neuroscience, as well as contemporary sociology.

## 3. Maurice Halbwachs’ renewed memory: the contributions of psychology and cognitive neuroscience

Over the past 20 years, psychologists and cognitive neuroscientists have developed a body of work devoted to the study of collective memory, relative to individual memory, including its cerebral substrates (Gagnepain et al., 2020). The rather imprecise theoretical field outlined by Halbwachs, which extended from collective memories to the social frameworks of memory, is now expressed in the form of two so-called *complementary* approaches. The first (top-down) approach takes social or cultural memories and social frameworks (social representations, narrative models, cultural patterns, etc.) as its starting point to study what individuals have retained and by what cognitive mechanisms they forget and remember, based on these social representations. The other (bottom-up) approach explores how individuals share knowledge and memories (Hirst and Manier, 2008). Mostly through social psychology experiments in the laboratory, it focuses on dyadic exchanges, with the overarching idea that what we observe at this local (interpersonal) level shapes what emerges at the global level (Hirst et al., 2018).

Regardless of which approach is preferred, there is no doubt here that *collective memory* refers to cognitive operations specific to individual systems of consciousness: only individuals remember, although they never remember alone (with reference to Halbwachs’ idea that we are never alone). It is as participants in interactions or as members of a group (formal or informal, large or small, real or experimental, etc.) that individuals make memories and refresh their memories. This can take place in different contexts, be it within the family (Reese and Fivush, 2008), at school, or in the workplace.

An essential contribution of psychology is that it goes beyond the metaphorical character of the collective memory concept, by substituting two operational concepts: shared memory and collaborative memory. The concept of *shared memory* refers to individuals as members of a group (shared values, social or collective identity), while the concept of *collaborative memory* refers more to individuals as participants in an interaction. This important distinction has been theorized and discussed relatively little in the literature.

### 3.1. Shared memory

Although the concept of collective memory has been the subject of much debate-and we can see from the above that it has multiple meanings, there seems to be unanimity over its operationalization through the concept of shared memory (Box 2).

BOX 2 **Collective memory as shared memory.**

Although “collective memory refers to the recollection of events shared by a group” (Roediger and Abel, 2015, p. 359), in practical terms it relates to “memories that individuals have as members of the groups to which they belong, whether small (family, school) or large (political party, nation)” (Roediger, 2021, p. 1388). This author emphasizes that collective memory is “held within individuals”, and goes on to underscore the *processual* (dialectic or dialogic) nature of collective memory, made up of conflicts and protests over how the past should be remembered by members of a group or community. “From this perspective, collective remembering is viewed as an active process that often involves contention and contestation among people rather than a static body of knowledge that they possess” (Wertsch and Roediger, 2008, p. 318). These reflections are pervaded by the concept of identity, be it personal or social. For Zaromb et al. (2014, p. 383), “a collective memory is a representation of the past that is shared by members of a group”, while according to Yamashiro and Roediger (2021, p. 311), “collective memories in the psychological sense are shared memories held individually by members of a group that pertain to their collective identity”. As for Wertsch and Jäggi (2022, p. 149), “collective memory can generally be defined as an account of the past that is shared by members of a group and is part of their identity project.”

The notion of *shared narrative* is also used by social scientists (e.g., Knapp, 1989). It is undoubtedly more accurate and exhaustive than the terms *shared memories* and *shared recollections*, which refer to singular and emblematic moments and do not constitute a full and complete memory. Moreover, even if these narratives are shared, their often polyphonic (or multivocal) character allows individuals to simultaneously retain multiple and contradictory points of view (Smith, 2004). In the case of collective trauma, Hirschberger (2018) showed that the dissonance between historical crimes and the need to uphold a positive image of the group can be resolved not only through disidentification from the group, but also through “the creation of a new group narrative that acknowledges the crime and uses it as a backdrop to accentuate the current positive actions of the group” (p. 1). For both victims and perpetrators, making sense of trauma is an ongoing process that is continuously negotiated within and between groups (see, for example, Kosicki and Jasińska-Kania, 2007).

While the question of *self* (or identity construction) is central to work on individual memory, particularly around the concepts of autobiographical memory and projection into the future (e.g., Tulving, 1985; Conway, 2005; Eustache et al., 2016), it is also central to research on collective memory (Box 3).

BOX 3 **Collective memory and collective identities.**

Many researchers have studied the links between collective memory and national or community identities (Hirst and Fineberg, 2012; Meier, 2021; Bachleitner, 2022; Fischer and O’Mara, 2022). For example, Merck et al. (2020) focused on soccer fan communities. In the specific case of hooliganism, King (2001) showed how violence serves as a medium of communication that allows the group (e.g., hooligan gang) to self-constitute and its members to assert their solidarity with the group. Tileagă (2012), meanwhile, focused on the moral and political dilemmas of remembering communism in Romania. An important point to note is that as collective memories are shared memories, they vary across generations (Caron-Diotte et al., 2022) and differ according to whether the events are personally experienced or learned from historical sources, further emphasizing the link between these shared memories and individual memories. This difference was analyzed by Muller et al. (2016) in a case study of collective memory in Argentina.

This research on the psychology of collective memory is consistent with theses defended in the social sciences, including Booth (2008)’s work on how memory contributes to the construction of identities, and Assmann and Czaplicka (1995)’s research distinguishing *cultural memory* (directly linked to identity) from history. This distinction between history and memory, which was discussed by Halbwachs, is a central one in the literature (Crane, 1997; Assmann, 2008; Wertsch, 2008a). Whereas historical science claims to be universal, exhaustive and impartial, collective memory always exists in the plural and is highly selective. History separates the past from the present and the future, whereas memory links them together. Memory always operates in the present: it is a continual rewriting of the past in the present for future use. Both sociologists and psychologists agree on this point (Schwartz, 1991; Coser, 1992; Wertsch, 2008b; Borer, 2010; French, 2012; Adams and Edy, 2021; Bachleitner, 2022).

At the intersection of research on shared memory and collaborative memory, a number of recent studies have focused on the role of communication in collective memory, where even conversation is regarded as an act of memory (Cyr and Hirst, 2019). Hirst and Echterhoff (2008, 2012), for instance, studied the role of conversations in the formation of collective memories, while other laboratory experiments have investigated how the temporal dynamics of conversations shape the formation of collective memories (Momennejad et al., 2019). Adopting the bottom-up approach defined earlier, Hirst and Coman (2018) showed that through communicative acts of memorization, memories can be shared within small groups and gradually expanded to the level of complex social networks to form a collective memory. As Hirst et al. (2018) aptly noted, the bottom-up approach treats the study of collective memory as an *epidemiological project*, seeking to understand why some memories spread throughout a group or community and others do not.

### 3.2. Collaborative memory

Collective memory is also operationalized through the concept of collaborative memory, with the focus on the social transmission and social contagion of memory (Roediger et al., 2001; Box 4).

BOX 4 **Collective memory as collaborative memory.**

Several researchers have explored the role of collaboration in collective memory (Harris et al., 2008; Barber et al., 2012; Maswood et al., 2019). False memories have been found to be particularly contagious (Choi et al., 2017). Rajaram and Pereira-Pasarin (2010) highlighted completely counterintuitive effects (e.g., forgetting and memory errors) of collaboration on memory. As for the cognitive mechanisms associated with the costs and benefits of collaboration on memory, Weldon and Bellinger (1997) provided a good description of *collaborative inhibition*, whereby collaborative groups remember fewer items than nominal groups. Yamashiro and Hirst (2020) studied the role of central speakers (politicians, journalists, etc.) in the formation of collective memory, and showed to what extent and under what conditions these speakers can reshape the memories (and forgetting) of a set of listeners. Mutlutürk et al. (2022) recently questioned the stability of collective memory representations and looked at changes in their organization, in terms of the roles of transformative events (e.g., a military coup) and sociopolitical identities.

All these studies highlighted an essential characteristic of collective memory, namely its malleability (Caron-Diotte et al., 2022). Collective memory is imperfect and necessarily imprecise: it is a process of reconstruction made up of distortions and glaring inaccuracies. Despite the drawbacks associated with this malleability, Brown et al. (2012) argued that the reconstructive nature of memory allows for greater cognitive flexibility and supports the construction and maintenance of identities (individual and collective). The malleability of memory thus supports the formation of shared memories.

Another benefit of this empirical research in social psychology is that it focuses on a key function of collective memory: collective selective forgetting (Choi et al., 2017; Hirst and Coman, 2018) or amnesia in collectives (Anastasio, 2022). This function has also been extensively described in sociology (Bucholc, 2013). Vinitzky-Seroussi and Teeger (2010) emphasized the importance of using collective silences (overt or latent) to reinforce memory and forgetting within the group, in order to *manage one’s past*. In psychology, collective silences have also been studied in the case of traumatic memories (Kevers et al., 2016).

Another important point to note is the role of metacognition in collective memory (Lund and Russell, 2022), namely, the capacity for self-reflection required for social interactions, through which collective memories are formed. From this perspective, Adams and Edy (2021)’s detailed analysis of a process of temporal negotiation revealed that changing the end of a story entails rewriting it in its entirety (see also Olick and Levy, 1997). This research is not dissimilar to the *interactionist project* in sociology (Fine and Beim, 2007) and the study of negotiations of meaning about the past (and future) by interactors (Borer, 2010). However, there is a key difference at the methodological level: sociologists observe interactions *in situ* (in the field), whereas psychologists observe them *in vitro* (in the laboratory). This type of decontextualized observation has many practical and analytical advantages for research, but there is inevitably a gap between the cognitive mechanisms that have been identified and the sociocultural environment in which these mechanisms develop.

### 3.3. A blind spot: the cultural foundations of collective memory

As Rajaram (2022) aptly summarizes, one of the main issues for research in this bottom-up approach is the impact of culture, which constitutes a blind spot in some ways. It is here that the complementarity of the bottom-up and top-down approaches becomes clear (Box 5).

BOX 5 **The cultural foundations of collective memory.**

Wang (2008) studied the impact of the cultural context in which collective memory takes place, through a detailed analysis of the functional variations in collective memory between cultures. In a more recent study on the cultural foundation of human memory (Wang, 2021, p. 154), he outlined a model of a “culturally saturated mnemonic system” in which cultural elements constitute and condition various processes of remembering (representation, perceptual encoding, function, reconstruction, expression, and socialization). For their part, adopting a resolutely top-down approach that included the use of brain imaging, Gagnepain et al. (2020) demonstrated that the *collective schemas* of the Second World War portrayed on French national television shape the organization of the memories of visitors to the Caen Memorial. As Nelson (2003) had earlier pointed out, autobiographical memory is functionally and structurally linked to the use of collective narratives and cultural myths.

Despite the undeniable value of these innovative studies and the complementarity of their approaches, they have remained firmly within the confines of the laboratory. The cultural context has, of course, been taken into account, but often reduced to a few quantitative variables, for the purposes of the investigation. Moreover, on a more theoretical level, although collective memory has been operationalized through the concepts of shared memory and collaborative memory, the same cannot be said for *social framework*, which has been insufficiently problematized. The articulation between what takes place at the level of systems of consciousness and what takes place at the level of social systems clearly remains fragile.

It is on this point, it seems to us, that sociology today has the most to contribute to the scientific debate. It can draw on a range of theoretical resources (of varied origins) to support the founding intuitions of Durkheim and Halbwachs, thus obviating the need for metaphors or reifications. This is the theory of social systems (Parsons, 1951), which Niklas Luhmann brilliantly formalized and updated at the end of the 1990s. Curiously enough, whereas Halbwachs’ writings are at the heart of current debates, Luhmann’s writings continue to be totally ignored in the literature on memory studies (including sociology). We will therefore try to make up for this oversight.

*Systems theory* allows us to think of society as a *sui generis* reality: an autopoietic system [as defined by Varela (2017)] that produces and reproduces itself through communication. Social systems are communication systems, composed exclusively of communication operations (and not of individuals or human beings). For example, a group is not a gathering of human beings, from this perspective, but an intense sequence of communication events (self-referential and self-produced): a group is not defined by the *people of its members* but by the scope of its norms (Teubner, 1996). For Luhmann (2021), human beings must be considered part of the environment of society. They participate in the operations of communication, but do not communicate. Only communication (and thus society) communicates. We will use his new theoretical foundations to clarify the concepts of social memory and collective memory.

## 4. Contribution of contemporary sociology: concept of social memory

Within memory studies, social scientists generally use the term *social memory* as a synonym (or extension) of collective memory, rather than as a concept in its own right. However, a few historians favor the concept of social memory over collective memory, to emphasize the social contexts in which people shape group identities and debate their conflicting perceptions of the past (e.g., French, 1995). In psychology and neuroscience, the concept of collective memory is more readily used to study humans and their primate ancestors (Lee et al., 2017). By contrast, the concept of social memory is favored over collective memory for studying rodents (e.g., van der Kooij and Sandi, 2012; Dias et al., 2016; Smith et al., 2016; Okuyama, 2018; Lunardi et al., 2021).

### 4.1. Concept of social memory

In Luhmann’s sociology, the concept of social memory refers very precisely to a specific operation of social systems: the constant distinction that is made between forgetting and remembering within communication processes (Luhmann, 2012, p. 137). Forgetting is essential, as it frees up the system’s information processing capacities and opens it up to new operations, preventing the system from becoming clogged up “by coagulating the results of its previous observations” (Luhmann, 2021, p. 396), as psychologists and psychiatrists have observed in the case of posttraumatic stress disorder (Mary et al., 2020).

This continuous discrimination, characterized by both great rigidity and great flexibility, allows the social system to “constantly say what is what” (Boltanski, 2009). For the social system, “memory consists in the fact that, in any communication, certain statements about reality can be presupposed as known without having to introduce them into the communication and justify them. Memory is at work in all operations of the system of society, that is, in all communications” (Luhmann, 2012, pp. 91–92). It is the primary role of institutions within social systems (Searle, 1995; De Munck, 1999), comparable to *re-entry phenomena* within the central nervous system (Edelman and Tononi, 2000). Social memory operates continuously, selectively re-imprinting the system’s own states. Repetition produces forgetting and remembering, always in the present.

How does the memory of society and social subsystems function? Social memory is not collective memory (as defined by Halbwachs), where individuals remember roughly the same facts when they are exposed to the same social conditions: “social memory is by no means constituted by the traces left by the communication in the individual systems of consciousness. It is, on the contrary, a proper performance of social systems, of communicative operations, with their own recursivity” (Luhmann, 2021, pp. 398–399). A social memory is formed by the simple fact that communication actualizes meanings, which are therefore already known to some extent. Repetition of the same references results in this continuous re-imprinting of usable meaning at the level of communication. Social memory does, of course, require the cooperation of systems of consciousness (as with any communication): social memory cannot function without individual systems of consciousness with memory. However, as Luhmann pointed out, social memory does not depend on what people remember or how they refresh their memory by participating in communication.

### 4.2. Operational autonomy of social memory

For Luhmann, every society depends on its own, self-produced memory. As oral communication is characterized by many communications taking place simultaneously, which cannot therefore be coordinated, ancient societies without writing necessarily used functional equivalents of writing (places, objects, sacred buildings) to refer to communication and provide memory for the system’s operations (preparing, supporting speech and listening). More specifically, in order to allow for the recognition of sameness or repetition, without having to rely on neurophysiological and psychological mechanisms, which are far too labile (e.g., Fanta et al., 2019), tribal or segmental societies (without writing) based social memories on sacred places, known spaces (topographical forms), and (sacred) objects and quasi-objects (symbolic forms), including rites, festivals, myths, and legends (Luhmann, 2021, pp. 439–440). The commemorative rites of the Warramunga, described by Durkheim, are a good example of this (see Section 2.1).

As the anthropologist Goody (2018) has eloquently demonstrated, the introduction of writing profoundly transformed the organization of society and allowed for the gradual formation of the main functional subsystems (religion, law, economy, politics). The introduction of writing thus constituted a key stage in the evolution of social memory: communication could henceforth be preserved in an autonomous way, independently of living memory and social interactions. It could reach absent ones. The social dimension gained in autonomy, compared with the material dimension. Until the modern era, as Luhmann noted, writing was above all regarded as a memory aid and a means of transport (transporting signs without having to move things): writing was a mnemonic device that profoundly modified the meaning of memory. The invention of writing allowed for the differentiation of a specifically social memory: writing was henceforth a memory that constituted itself, and people could now remember and forget more things than before. “Writing modifies the possibilities of developing a social memory independent of the neurophysiological and psychological mechanisms of individuals” (Luhmann, 2021, p. 211).

Printing emerged and developed as a technical infrastructure that could maintain and update a memory of society, separate from what individuals more or less remembered, and above all independent of the generational changing of the guard. In order to make this memory more widely available, public libraries, museums, and documentation centers were created. As Blouin (1999) showed, archivists play an essential role in the construction of a social memory: they ensure a complex function of mediation between what has survived and what we know.

Back in 1937, Talcott Parsons had already come to consider the cultural system as the maintenance (or memory) subsystem of society (Parsons, 1937). Modern society invented the concept of culture to designate its memory, and the cultural system was differentiated from the social system. As Luhmann suggested, the concept of culture allows modern society to self-adapt its memory to the requirements of the functional differentiation of society into autonomized subsystems. Culture is social memory: the filtering between forgetting and remembering, always in the present, and the link between past and future. As Pierre Bourdieu (1979) skillfully showed, the concept of *cultural capital* designates this accumulated past, which can be used in the present as a resource for the future. Schwartz (1982, 1991, 1997)’s work provides a very good illustration of this point (Box 6).

BOX 6 **The commemorative symbolism: a function of social memory.**

As a direct extension of Durkheim’s pioneering studies, Schwartz asked two essential questions: How does commemorative symbolism function? And how does society produce an autonomous memory of what individuals remember? These questions did not concern individuals or the memory traces left in individual systems of consciousness, but social systems‑in other words, the communication operations of functional systems of society, such as science, the press, and the arts (painting, engraving, sculpture, caricature, etc.). Schwartz was interested in communication operations that communicated themselves, without having to depend directly on individual and collective memories: an article referring to another article, a painting referring to a pamphlet or another painting, a caricature of an already famous portrait, and so on. These innumerable communications, which referred only to themselves and operated continuously, produced repetition, redundancy and, through the repetition of differences, evolved autonomously. In his first case study of the iconography of the Capitol, Schwartz showed that the meaning of historical events changed massively, as a result of changes in society (before and after the Civil War). However, contrary to Halbwachs’ claim, the past cannot literally be reconstructed in every generation in a wholly contingent manner, given the constraints of a *recorded history*: it can only be selectively exploited: “In the last 120 years, the commemoration of America’s origins has been enriched by the addition of many works of art. These acquisitions mean that the transformation in commemorative practice inheres not in the displacement of early figures but in the superimposition of more recent, and in many cases less heroic, men and events” (Schwartz, 1982, p. 396). This observation is in line with Durkheim’s theses on the evolutionary continuity of social memory, which contrasts with the lability of the cognitive processes behind collective memory. Two other case studies (one on the democratization of George Washington and the other on the Lincoln Day celebration) confirmed this important finding (see also Ghoshal, 2013). As Schwartz noted, social systems have set limits on the ability of successive generations to democratize Washington and, as a corollary, societal changes have determined how Washington’s original image is both revised and preserved. There is no need, therefore, for one theory to think about change and another to think about continuity, as a single theory can account for the evolution of memory at two distinct analytical levels: consciousness systems and social systems.

**Figure 1:**
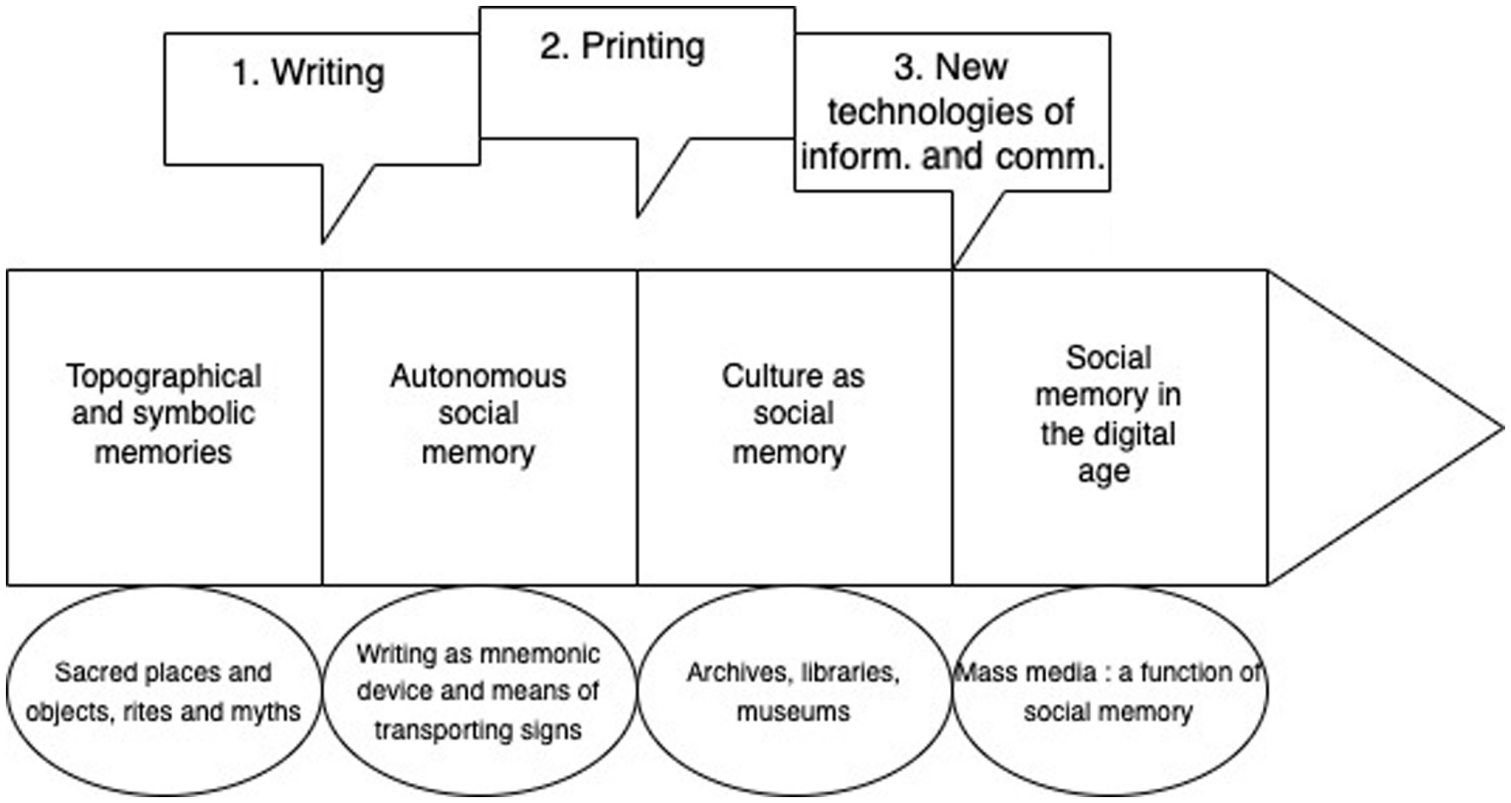
Formation and development of social memory: three historical stages. This figure represents the formation and the development of social memory, through three historical stages: writing, printing and new information, and communication technologies. Societies without writing used topographical and symbolic memories that depended heavily on living memory and human interactions. 1. Writing has enabled the formation of an autonomous social memory, independent of the living: it is a memory aid and a novel means of transport (of signs, not things). 2. Printing enabled this autonomous social memory to be disseminated via archives, libraries and museums: with the advent of modernity, the term culture gradually came to designate the memory of society. 3. New information and communication technologies enable communications operations to be disseminated globally and instantaneously: with technology, the mass media fulfill the function of social memory on a planetary scale within modern society.

While the advent of writing and the development of printing constituted two important stages in the development of an autonomous social memory of the living, we are now embarking on a third phase, with the emergence of new communication technologies. Digital technologies are emerging as the new social conditions for memory (Hui, 2017; Box 7)). Within modern society, the mass media perform an important selection and sorting function on a global scale, guiding and framing the archiving of cultural products and memories (Figure 1).

BOX 7 **Social memory in the digital age.**

Yasseri et al. (2022) attempted to assess the impact of the digital transformation of society on the way we produce, communicate, and acquire information. How do digital tools and data (as traces) transform collective memories? To answer this question, García-Gavilanes et al. (2017) analyzed viewership statistics for Wikipedia articles on aircraft crashes and devised a quantitative model to explain the flow of viewership from a current event to past events (based on their similarity). They discovered previously unknown cascade effects: on average, the secondary flow of attention to past events, generated by memory processes, was larger than the primary flow of attention to the current event. Social memories therefore serve as *attractors* for individual and collective memories. Other researchers have explored the decay of collective memory (in the sense here of shared memory; e.g., Frank, 2019) over time and, as a corollary, the permanence of cultural objects such as video games (Mendes et al., 2022) or popular music (Spivack et al., 2019). Some have simply described this temporal decay in the amount of attention paid to cultural products (articles, patents, movies, songs, biographies), in the form of a universal bi-exponential curve (Candia et al., 2019). Others have attempted to predict the decay of collective memory (Coman, 2019): if cultural products have a life of their own, the pattern of steady decline could be due to the way cultural products are discussed within the community and archived as cultural memories.

### 4.3. Mass media: a function of social memory

Modern society can be described as a functionally differentiated society (Luhmann, 1999), within which each subsystem (law, science, arts, politics, economy, etc.) takes on part of the social complexity, processing it according to its own indecipherable code. This functional differentiation of society gives rise to increasingly autonomous subsystems with no central steering body to supervise the subsystems. Each communication subsystem is therefore regulated by a code and has its own memory and specific basis for sorting between forgetting and remembering: law has a jurisprudence that makes it possible to forget the details of each case^1^; science has repertoires of publications that make it possible to forget the wanderings of each research study; education has selection criteria that make it possible to forget students’ social origins; and the monetary economy has an abstract mode of counting value designed to forget the origin of payments (which the legal system must sometimes correct), in order to facilitate transactions.

The mass media constitute a specific functional subsystem of modern society, an important *institution of meaning* at the global level that serves as a frame of reference and fulfills an essential function of sorting between forgetting and remembering. According to Luhmann (2006), each system is defined by the difference between itself and the environment, with its code constituting the unit of difference. In the case of the mass media subsystem, the code distinguishes between information and noninformation. Producing information is an internal operation for every system, and the reality of the mass media is always a duplication of reality. The mass media subsystem performs two essential functions (Luhmann, 2012): (1) semantic securitization: constantly saying what is what, at the level of the society-world (re-entry of the difference between forgetting and remembering); and (2) critical awareness: constantly sensitizing the social system (to criticism, surprises, news, novelty, deviance, conflict, provocation, etc.), keeping it constantly on the alert, and exposing it to challenge (like an immune system).

The mass media provide an often dramatic staging of events: surprise, local reference, quantities, and conflictuality (transgression of norms) all constitute *information selectors*. In other words, what constitutes the programming of expectations within the mass media system, what can be expected as being information, and what must remain without value. The mass media system extracts events from the world, defines the reference frame, fabricates the social reality, and internally produces *information*. Other social subsystems may or may not be sensitized by these media events and react to them, each in its own way, according to a specific timeframe: the economic system by increasing prices, the political system by taking collectively binding decisions, the legal system by stabilizing normative expectations, the scientific system by developing new research programs, and so on.

To sum up, at the risk of repeating ourselves: social memory is not collective memory. Social memory emerges from communication operations specific to social systems, whereas collective memory emerges from the operations of individual systems of consciousness when people participate in communication operations in *social life* (as members of groups or in interaction situations). However, we have yet to address the important issue of their articulation, in other words, the *contagious co-evolution* of collective memories and the social structure (Lee et al., 2010). In our opinion, Cicourel (2015)’s *fusion* model is not entirely convincing, as it collapses quite distinct levels of analysis (Barnier and Sutton, 2008; Sutton, 2008).

## 5. Collective memory and social memory: two structural coupling mechanisms

The question of how collective and social memories interact brings us back to the more general theoretical question of how systems of consciousness that are operationally closed interact with social systems that are also closed. A theory of intersystemic mediations has yet to be constructed. Fortunately, the concepts of interpenetration in sociology (Parsons, 2004; Luhmann, 2010) and structural coupling in neurobiology (Maturana and Varela, 1994) allow us to move forward in this direction (Box 8).

BOX 8 **The concepts of interpenetration and structural coupling.**

*Interpenetration* is a key concept for the analysis of intersystemic relations. If two interpenetrating systems remain environments for each other, it is because the complexity made available to one is always elusive for the other (noise). According to Luhmann, the evolution of systems (social and psychic) follows the principle of *order from noise*: each system provides the other with just enough disorder for an order to gradually emerge from random events. Thus, social systems are formed on the basis of the noise that individual systems of consciousness produce in their attempts to communicate (Luhmann, 2010, p. 268). The concept of structural coupling between system and environment (Varela, 2017, p. 259) usefully complements this notion, as it refers to a process that *digitalizes analogical relations* (as defined by Baetson, 1980). As the environment (composed of other systems) operates simultaneously with the system, there are initially only analogical (i.e., parallel) relations. The challenge is therefore to transform these analogical relations into digital ones, by means of binary coding, so that the environment can act on the system (e.g., the eye or the ear and the corresponding operations in the brain).

**Figure 2:**
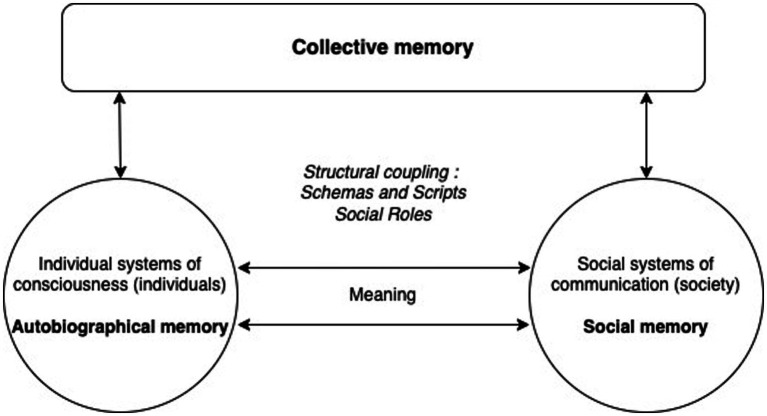
Structural coupling: schemas, scripts, and social roles. At the top of the figure, collective memory appears as an emergent phenomenon, between individual systems of consciousness (individuals) and social systems (society). Collective memory is a specific operation of individual consciousness when it participates in or connects to the communication that constitute society, either as member of social group (shared memory), or as participant in social interaction (collaborative memory). The circle on the left represents individual consciousness: autobiographical memory corresponds to the operations of selection (sorting between forgetting and remembering) of an individual system of consciousness. The circle on the right represents social systems: social memory corresponds to the operations of selection (sorting between forgetting and remembering) of society as a communication system. The double arrow linking the two circles represents the universal medium of all psychic and social systems, meaning: the result of the joint evolution of individuals and society. The semantic component of autobiographical memory is thus directly linked to social memory, to the multiple selections of meaning produced and reproduced by communication operations. In the center of the figure, schemas and scripts, as well as social roles, make possible the structural coupling between consciousness and communication, thanks to the common medium of meaning.

Let us begin by recalling that it is language that allows for a permanent structural coupling between systems of consciousness and systems of communication (Luhmann, 2021). All communication is structurally coupled with consciousness: it is totally dependent on it (although consciousness is neither the subject nor the medium of communication). The bifurcation of the communication code that is language allows consciousness to opt for either side of the form (in the sense of Spencer-Brown, 1969): all communication offers the double possibility of being accepted or refused, and any meaning can be expressed in a positive or negative way. In addition to language, we identify at least two very specific mechanisms of structural coupling (between individual systems of consciousness and social systems) arranged in a more labile way and capable of learning (see Halbwachs): schemas/scripts and social roles (Figure 2).

A *schema* (or frame, script, stereotype, mental map, etc.) is a combination of meanings that allows society and individual systems of consciousness to constitute a memory that can forget almost all its operations but nevertheless retain some of them in a schematized form (Luhmann, 2012), be it temporal, attributional, preferential, perceptual, narrative, or decisional. Schemas are the instruments of forgetting (and learning): they are not images, but rules for performing (or repeating) operations (not the image of the circle, as Kant made clear, but the rules for drawing it), and can refer to either things or people. Decades after Bartlett (1932)’s pioneering work, they have become a prime focus of research in memory studies (Beim, 2007; Tse et al., 2007; Van Swol, 2008; Wertsch, 2008a,b; Wertsch and Jäggi, 2022). As Abelson (1981) notes, *scripts* are special cases of schemas with stereotyped temporal successions (e.g., buying a ticket before entering a movie theater). The concept of *cultural script* (Green, 2004) is central here. We can hypothesize that the structural coupling between mass media communication and individual systems of consciousness uses and even generates scripts, particularly causal ones, about the environment, terrorism, the pandemic, and so on (see, for example, Luo et al., 2022). The mass media system provides individual systems of consciousness with scripts that allow them to organize their memories (i.e., to forget almost everything about the experience and retain only a few elements of it in a highly simplified form). However, the mass media are not the only subsystem of society in which consciousness systems participate (here, as spectators), as the other subsystems also rely on participation (e.g., in the political system as citizens, in the economic system as consumers, in the educational system as learners, in the legal system as plaintiffs).

The concept of *social role* is very useful for understanding a second coupling mechanism between individual systems of consciousness and social systems. Parsons (2004) defined this concept as a system of *anticipations* (instrumental, expressive, and moral) that link the performer of the role to those for whom it is performed. According to him, social roles constitute fundamental zones of interpenetration between the social system and the personality of the individual (Parsons, 1963). To take part in communication operations in social life events, individual systems of consciousness use social roles, just as actors use *masks* to go on stage (Strauss, 1992). It is through the prism of a role that an individual consciousness can connect to society, to functional systems (as a client, beneficiary, pupil, parent, etc.), to multiple organizations (as a member), and to interaction systems (as a participant). Social roles therefore condition the formation of memories (individual and collective), and modulate the operations of selection and sorting between forgetting and remembering. If memory is closely linked to self (Conway, 2005), it is because the social roles through which identities are formed (Mead, 2006) frame and orient the cognitive operations of individual systems of consciousness.

As Luhmann (2021) noted, in order to participate in all subsystems of society, individuals must take into account the specific communication codes of each subsystem and be able to change their structural couplings with these functional systems at any time (i.e., change roles). Fortunately, they can rely on highly differentiated, symbolically generalized communication media added to everyday language, which they more or less master in the course of their socialization, such as money in the economic system, truth in the scientific system, power in organizations, and love in romantic and family relationships. The binary coding of these communication media (truth/nontruth, governing/governed, right/nonright, information/noninformation, etc.) fulfills two essential purposes: guaranteeing the autopoiesis of communication (each side communicates with the other), and facilitating the structural coupling with systems of consciousness (opting for one side or the other). It should also be noted that each communication medium regulates a single symbiotic mechanism (e.g., sexuality for love, need for money, perception for truth, physical force for power; Luhmann, 2021). Through specific codes of communication, social roles therefore make it possible to include the organism (body) in this intersystemic relationship between consciousness and communication-in other words, to connect bodily memories to collective and social memories. In the case of the musicians that Halbwachs studied, it was through a specific social role (e.g., first violin or double bass player) that a collective memory was formed, at the intersection of organic life, individual consciousness, and communication (society).

## 6. Conclusion

Now we have reached the end of this review of the *wide-angle* literature on collective memory, we feel it is important to return to the conceptual clarifications that have allowed us to map out the relevant notions, in order to find our way in a teeming, chaotic and diversified field of research, and above all to facilitate multidisciplinary exchanges within memory studies.

Recent research on collective memory has identified four key issues pertaining to this multidisciplinary research topic: (1) *collective memory* refers to the memories of individuals either as members of a group or community (*shared memory*) or as participants in an interaction (*collaborative memory*); (2) collective memories are formed through processes of identity construction (*Self*); (3) insofar as it involves sorting between remembering and forgetting, the essential function of collective memory is forgetting; and (4) unlike history, which separates the past from the present and the future, collective memory connects them, always operating in the present as a continual rewriting of the past for future use.

It is important to make a distinction between collective memory and *social memory*: the former is a specific operation of individual systems of consciousness, while the latter is a performance of social systems, and of self-referential and self-produced operations of communication. Writing, printing, and the new communication technologies can be regarded as three key stages in the formation and development of an autonomous social memory that is independent of living individuals and of social interactions. As we have shown, the mass media in our modern era fulfill an essential function of social memory, by sorting between forgetting and remembering, on the scale of a globalized society.

When thinking about the articulation between collective memory and social memory, the concept of structural coupling allows us to identify two mechanisms by which individual systems of consciousness and social systems can interact and be mutually sensitized: schemas and scripts, and social roles. Schemes and scripts are the instruments of forgetting (and learning) that social systems (e.g., mass media) offer to individual systems of consciousness so that they can participate in communication operations. As for social roles, they allow consciousness to connect with society, functional systems, multiple organizations, and interaction systems. It is therefore through social roles that individual systems of consciousness (i.e., individuals) can sort between forgetting and remembering (at both encoding and retrieval).

Social roles therefore condition the formation of both individual and collective memories, and seem a particularly fruitful avenue for cross-disciplinary research on memory. To further illustrate our theoretical argument, let us take the example of the work carried out as part of the 13/11 transdisciplinary program (Eustache and Peschanski, 2022). In this long-term study of collective memories of the 2015 Paris attacks, an interesting hypothesis would be to empirically test the influence of social roles (survivor, police officer, bereaved, witness, etc.) in the construction of memories and narratives of the event. On a methodological level, the tools of textometry seem particularly well-suited to reveal, within a corpus of nearly 1,000 individual testimonies, the lexical fields specific to the different social roles that people were led to play in this social drama, and to see to what extent these specific vocabularies modulate the sorting between forgetting and remembering, and structure the collective memories of November 13. From this perspective, the role of the television viewer of the attack is particularly interesting for studying the influence of mass media (as the social memory of the event) on the formation of individual and collective (shared) memories: how do the scripts provided by the social system of mass media act on the collective memory of television viewers (Orianne, 2023)?

In clinical terms, exploring the coherence between individual and collective memory, for a singular individual, is particularly relevant in Post-Traumatic Stress Disorder (PTSD), enabling a better understanding of risk and resilience factors. The memory distortions at the heart of this disorder have a very specific profile, combining hypermnesia of certain emotional and perceptual aspects linked to the traumatic event with more or less marked amnesia of contextual aspects. The autobiographical memory of these patients is impaired, as evidenced by their difficulty in distancing themselves from the traumatic event and making it lose its immediacy. Patients tend to regard their trauma as a major autobiographical event, characterizing them in the first instance, but poorly integrated into their overall life course. In addition, altered self-image, dominated by negative perceptions, guides the nature of recalled memories. Emotional memory disorders lie at the heart of PTSD, and therapies aim to reduce the emotional burden of traumatic memories, making them “acceptable.” The existence of a reassuring context around the patient is also a protective factor.

This context involves the family and the workplace, but must also extend to the social environment. In this respect, we hypothesize that the collective memory attached to a traumatic event, all the more so in the case of a large-scale event, will play a major role in the individual’s memory. If this collective memory is in phase with the individual’s own memory, it will act as a catalyst in consolidating his or her memories, enabling them to become acceptable. Beyond this, it will encourage the development of resilience mechanisms, as the social framework supports the reconstruction mechanisms. If, on the other hand, these two forms of memory develop in a disordered, even antagonistic way, they will both be weakened, with harmful effects.

This reading of the joint construction, discordant or not, of different strata of individual and collective memories, could find applications in various situations that place the individual in an existential rupture. For example, memory disorders have been described in many diseases that have no direct repercussions on brain function, even before pharmacological treatments have been introduced. Breast cancer in particular has been studied in this context (Giffard et al., 2013). These memory disorders can be understood, in part, as the result of a psychosociological upheaval linked to a change in status, leading to a mismatch between individual (and collective) memory and social environment: a person who is integrated into active social life becomes a sick person, with other constraints, other concerns, and a different perception by others. This theoretical framework also opens up new avenues of reflection for patient care, particularly in terms of how those around the patient - caregivers, helpers but also the wider social environment - must adapt to the modified but constantly evolving existential trajectory of a singular patient.

This approach can also find relevant developments in patients with memory pathology (such as Alzheimer’s disease or amnesic syndrome). In these cases, memory disorders are severe, with retrograde amnesia going far back into the past. Patients may or may not experience a discrepancy between their day-to-day experience (e.g., living in a hospital or nursing home) and the memory of their previous environment to which they remain attached, a discrepancy that can extend over several decades. Here again, the distortions in autobiographical memory between day-to-day memory and the “social framework” are a means of understanding memory disorders and, more broadly, cognitive and behavioral disorders, and a potential guide to patient care.

Memory as studied by psychologists and memory as studied by historians and sociologists are not two separate concepts. The analogies described in both cases do not refer to metaphors, but underline the need for a trans-disciplinary approach, which is still largely to be invented, but whose theoretical importance and multiple applications, particularly in mental health and in a country’s memorialization policy, are now well identified.

## Author’s note

The quotations from Halbwachs and Luhmann were translated by the authors. The page numbers correspond to the French editions of their works, as indicated in the references.

## Author contributions

J-FO and FE contributed to the design, structure, content of the conceptual analysis, and writing the final draft. J-FO prepared the first draft. All authors contributed to the article and approved the submitted version.

## Footnotes

^1^On the reciprocal links between law and memory see, for example, Savelsberg and King (2007).

## References

[ref1] Abelson R. (1981). Psychological status of the script concept. Am. Psychol. 36, 715–729. doi: 10.1037/0003-066X.36.7.715, PMID: 26492591

[ref2] Adams T. Edy J. A. (2021). How the past becomes the past: the temporal positioning of collective memory. Br. J. Sociol. 72, 1415–1429. doi: 10.1111/1468-4446.12881, PMID: 34350979

[ref3] Anastasio T. J. (2022). Deriving testable hypotheses through an analogy between individual and collective memory. Prog. Brain Res. 274, 99–128. doi: 10.1016/bs.pbr.2022.06.001, PMID: 36167453

[ref4] Apfelbaum E. (2010). “Halbwachs and the social properties of memory” in Memory: Histories, theories, debates. eds. Radstone S. Schwartz B. (New York: Fordham University Press), 77–92.

[ref5] Assmann A. (2008). Transformations between history and memory. Soc. Res. 75, 49–72. doi: 10.1353/sor.2008.0038

[ref6] Assmann J. Czaplicka J. (1995). Collective memory and cultural identity. New German Critique 65, 125–133. doi: 10.2307/488538

[ref7] Bachleitner K. (2022). Collective memory and the social creation of identities: linking the past with the present and future. Prog. Brain Res. 274, 167–176. doi: 10.1016/bs.pbr.2022.07.002, PMID: 36167448

[ref9001] Baetson G. (1980). Vers une écologie de l’esprit. Paris: Seuil.

[ref9002] Barber S. J. Rajaram S. Fox E. B. (2012). Learning and remembering with others: the key role of retrieval in shaping group recall and collective memory. Soc. Cogn. 30, 121–132. doi: 10.1521/soco.2012.30.1.121, PMID: 25431516 PMC4244002

[ref8] Barnier A. J. Sutton J. (2008). From individual to collective memory: theoretical and empirical perspectives. Memory 16, 177–182. doi: 10.1080/09541440701828274, PMID: 18324545

[ref9] Bartlett F. (1932). Remembering: A study in experimental and social psychology. New York: Cambridge University Press.

[ref10] Beim A. (2007). The cognitive aspects of collective memory. Symb. Interact. 30, 7–26. doi: 10.1525/si.2007.30.1.7, PMID: 37669934

[ref11] Blouin F. X. (1999). Archivists, mediation, and constructs of social memory. Arch. Issues 24, 101–112.

[ref12] Boltanski L. (2009). De la critique. Paris: Gallimard.

[ref13] Booth W. J. (2008). The work of memory: time, identity, and justice. Soc. Res. 75, 237–262. doi: 10.1353/sor.2008.0040, PMID: 37119537

[ref14] Borer M. I. (2010). From collective memory to collective imagination: time, place, and urban redevelopment. Symb. Interact. 33, 96–114. doi: 10.1525/si.2010.33.1.96

[ref15] Bourdieu P. (1979). Les trois états du capital culturel. Actes de la recherche en sciences sociales 30, 3–6. doi: 10.3406/arss.1979.2654

[ref16] Brown A. D. Kouri N. Hirst W. (2012). Memory's malleability: its role in shaping collective memory and social identity. Front. Psychol. 3:257. doi: 10.3389/fpsyg.2012.00257, PMID: 22837750 PMC3402138

[ref17] Bucholc M. (2013). On the potential of Norbert Elias’s approach in the social memory research in central and Eastern Europe. Polish Sociol. Rev. 183, 317–334.

[ref9003] Candia C. Jara-Figueroa C. Rodriguez-Sickert C. Barabási A. L. Hidalgo C. A. (2019). The universal decay of collective memory and attention. Nat. Hum. Behav. 3, 82–91. doi: 10.1038/s41562-018-0474-5, PMID: 30932052

[ref18] Caron-Diotte M. de la Sablonnière R. Sadykova N. (2022). The malleability of collective memories: one year after the tulip revolution in Kyrgyzstan. Br. J. Soc. Psychol. 61, 192–213. doi: 10.1111/bjso.12476, PMID: 34142379

[ref19] Choi H. Y. Kensinger E. A. Rajaram S. (2017). Mnemonic transmission, social contagion, and emergence of collective memory: influence of emotional valence, group structure, and information distribution. J. Exp. Psychol. Gen. 146, 1247–1265. doi: 10.1037/xge0000327, PMID: 28594190

[ref20] Cicourel A. V. (2015). Collective memory, a fusion of cognitive mechanisms and cultural processes. Rev. Synth. 136, 309–328. doi: 10.1007/s11873-014-0258-7, PMID: 25078868

[ref9004] Coman A. (2019). Predicting the decay of collective memory. Nat. Hum. Behav. 3, 18–19. doi: 10.1038/s41562-018-0480-7, PMID: 30932045

[ref21] Coman A. Brown A. D. Koppel J. Hirst W. (2009). Collective memory from a psychological perspective. Int. J. Polit. Cult. Soc. 22, 125–141. doi: 10.1007/s10767-009-9057-9

[ref22] Confino A. (1997). Collective memory and cultural history: problems of method. Am. Hist. Rev. 102, 1386–1403. doi: 10.2307/2171069, PMID: 24005644

[ref23] Conway M. A. (2005). Memory and the self. J. Mem. Lang. 53, 594–628. doi: 10.1016/j.jml.2005.08.005, PMID: 37742906

[ref24] Coser L. A. (1992). The revival of the sociology of culture: the case of collective memory. Sociol. Forum 7, 365–373. doi: 10.1007/BF01125050, PMID: 19999633

[ref25] Crane S. A. (1997). Writing the individual Back into collective memory. Am. Hist. Rev. 102, 1372–1385. doi: 10.2307/2171068

[ref26] Cyr T. G. Hirst W. (2019). Reflections on conversations and memory. Top. Cogn. Sci. 11, 831–837. doi: 10.1111/tops.12437, PMID: 31385446

[ref27] De Munck J. (1999). L’institution sociale de l’esprit. Paris: PUF.

[ref28] Dias T. L. Golino H. F. de Oliveira V. E. M. Moraes M. F. D. Pereira G. S. (2016). C-Fos expression predicts long-term social memory retrieval in mice. Behav. Brain Res. 313, 260–271. doi: 10.1016/j.bbr.2016.07.030, PMID: 27449201

[ref29] Durkheim E. (1950), Les règles de la méthode sociologique. Paris: PUF.

[ref30] Durkheim E. (1960), Les formes élémentaires de la vie religieuse. Paris: PUF.

[ref9101] Ebbinghaus H. (2010). “La mémoire,” in Recherches de psychologie expérimentale. Paris: L’Harmattan.

[ref31] Edelman G.M. Tononi G. (2000). Comment la matière devient conscience. Paris: Odile Jacob.

[ref32] Eustache F. Peschanski D. (2022). Toward new memory sciences: the programme 13-Novembre. Prog. Brain Res. 274, 177–201. doi: 10.1016/bs.pbr.2022.07.003, PMID: 36167449

[ref33] Eustache F. Viard A. Desgranges B. (2016). The MNESIS model: memory systems and processes, identity and future thinking. Neuropsychologia 87, 96–109. doi: 10.1016/j.neuropsychologia.2016.05.006, PMID: 27178309

[ref34] Fanta V. Šálek M. Sklenicka P. (2019). How long do floods throughout the millennium remain in the collective memory? Nat. Commun. 10:1105. doi: 10.1038/s41467-019-09102-3, PMID: 30846690 PMC6405947

[ref35] Fine G. A. Beim A. (2007). Introduction: interactionist approaches to collective memory. Symb. Interact. 30, 1–5. doi: 10.1525/si.2007.30.1.1

[ref36] Fischer V. O'Mara S. M. (2022). Neural, psychological, and social foundations of collective memory: implications for common mnemonic processes, agency, and identity. Prog. Brain Res. 274, 1–30. doi: 10.1016/bs.pbr.2022.07.004, PMID: 36167445

[ref9005] Frank S. A. (2019). How to understand behavioral patterns in big data: the case of human collective memory. Behav. Sci. (Basel) 9:40. doi: 10.3390/bs9040040, PMID: 31014045 PMC6523660

[ref38] French B. M. (2012). The semiotics of collective memories. Annu. Rev. Anthropol. 41, 337–353. doi: 10.1146/annurev-anthro-081309-145936, PMID: 37154121

[ref37] French S. A. (1995). What is social memory? South. Cult. 2, 9–18. doi: 10.1353/scu.1995.0049, PMID: 37743415

[ref39] Gagnepain P. Vallée T. Heiden S. Decorde M. Gauvain J. L. Laurent A.. (2020). Collective memory shapes the organization of individual memories in the medial prefrontal cortex. Nat. Hum. Behav. 4, 189–200. doi: 10.1038/s41562-019-0779-z, PMID: 31844272

[ref9006] García-Gavilanes R. Mollgaard A. Tsvetkova M. Yasseri T. (2017). The memory remains: understanding collective memory in the digital age. Sci. Adv. 3:e1602368. doi: 10.1126/sciadv.1602368, PMID: 28435881 PMC5381953

[ref40] Gedi N. Elam Y. (1996). Collective memory — what is it? Hist. Mem. 8, 30–50.

[ref902] Ghoshal R. A. (2013). Transforming collective memory: mnemonic opportunity structures and the outcomes of racial violence memory movements. Theory Soc. 42, 329–350.

[ref41] Giffard B. Viard A. Dayan J. Morel N. Joly F. Eustache F. (2013). Autobiographical memory, self, and stress-related psychiatric disorders: which implications in cancer patients? Neuropsychol. Rev. 23, 157–168. doi: 10.1007/s11065-013-9233-6, PMID: 23640242

[ref42] Goody J. (2018). La logique de l’écriture. L’écrit et l’organisation de la société. Paris: Armand Colin.

[ref43] Green A. (2004). Individual remembering and “collective memory”: theoretical presuppositions and contemporary debates. Oral Hist. 32, 35–44.

[ref44] Halbwachs M. (1925). Les cadres sociaux de la mémoire. Paris: Félix Alcan.

[ref9007] Halbwachs M. (1939). La mémoire collective chez les musiciens. Revue philosophique, 136–165.

[ref45] Halbwachs M. (1947). La mémoire collective et le temps. Cah. Int. Sociol. 4, 45–65.

[ref46] Halbwachs M. (1950). La mémoire collective. Paris: PUF.

[ref9008] Harris C. B. Paterson H. M. Kemp R. I. (2008). Collaborative recall and collective memory: what happens when we remember together? Memory 16, 213–230. doi: 10.1080/09658210701811862, PMID: 18324548

[ref47] Hirschberger G. (2018). Collective trauma and the social construction of meaning. Front. Psychol. 9:1441. doi: 10.3389/fpsyg.2018.01441, PMID: 30147669 PMC6095989

[ref48] Hirst W. Coman A. (2018). Building a collective memory: the case for collective forgetting. Curr. Opin. Psychol. 23, 88–92. doi: 10.1016/j.copsyc.2018.02.002, PMID: 29459336

[ref49] Hirst W. Echterhoff G. (2008). Creating shared memories in conversation: toward a psychology of collective memory. Soc. Res. 75, 183–216. doi: 10.1353/sor.2008.0061

[ref50] Hirst W. Echterhoff G. (2012). Remembering in conversations: the social sharing and reshaping of memories. Annu. Rev. Psychol. 63, 55–79. doi: 10.1146/annurev-psych-120710-100340, PMID: 21961946

[ref9010] Hirst W. Fineberg I. A. (2012). Psychological perspectives on collective memory and national identity: the Belgian case. Memory Stud. 5, 86–95. doi: 10.1177/1750698011424034

[ref51] Hirst W. Manier D. (2008). Towards a psychology of collective memory. Memory 16, 183–200. doi: 10.1080/09658210701811912, PMID: 18324546

[ref52] Hirst W. Yamashiro J. K. Coman A. (2018). Collective memory from a psychological perspective. Trends Cogn. Sci. 22, 438–451. doi: 10.1016/j.tics.2018.02.010, PMID: 29678236

[ref53] Hui Y. (2017). “On the synthesis of social memories” in Memory in motion: Archives, technology and the social. eds. Blom I. Lundemo T. Røssaak E. (Amsterdam: Amsterdam University Press), 307–326.

[ref54] Hutton P. H. (1988). Collective memory and collective mentalities: the Halbwachs-Ariés connection. Historical Reflections/Réflexions Historiques 15, 311–322.

[ref55] Kansteiner W. (2002). Finding meaning in memory: a methodological critique of collective memory studies. Hist. Theory. 41, 179–197. doi: 10.1111/0018-2656.00198

[ref56] Kevers R. Rober P. Derluyn I. De Haene L. (2016). Remembering collective violence: broadening the notion of traumatic memory in post-conflict rehabilitation. Cult. Med. Psychiatry 40, 620–640. doi: 10.1007/s11013-016-9490-y, PMID: 27021343

[ref9011] King A. (2001). Violent pasts: collective memory and football hooliganism. Sociol. Rev. 49, 568–585. doi: 10.1111/1467-954X.00348

[ref57] Knapp S. (1989). Collective memory and the actual past. Representations 26, 123–149. doi: 10.2307/2928526, PMID: 31965905

[ref58] Kosicki P. H. Jasińska-Kania A. (2007). Guest editors’ introduction: aggressors, victims, and trauma in collective memory. Int. J. Sociol. 37, 3–9. doi: 10.2753/IJS0020-7659370100

[ref59] Laikhuram P. (2022). Collective memory: metaphor or real? Integr. Psychol. Behav. Sci. 57, 189–204. doi: 10.1007/s12124-022-09683-7 35325400

[ref60] Lee E. D. Daniels B. C. Krakauer D. C. Flack J. C. (2017). Collective memory in primate conflict implied by temporal scaling collapse. J. R. Soc. Interface 14:20170223. doi: 10.1098/rsif.2017.0223, PMID: 28878031 PMC5635334

[ref61] Lee S. Ramenzoni V. C. Holme P. (2010). Emergence of collective memories. PLoS One 5:e12522. doi: 10.1371/journal.pone.0012522, PMID: 20824141 PMC2931705

[ref62] Llobera J. R. (1995). Halbwachs, Nora and “history” versus “collective memory”: a research note. Durkheimian Studies/Études Durkheimiennes 1, 35–44.

[ref63] Luhmann N. (1999). Politique et complexité. Paris: Cerf.

[ref64] Luhmann N. (2006). System as difference. Organization 13, 37–57. doi: 10.1177/1350508406059638

[ref65] Luhmann N. (2010). Systèmes sociaux. Esquisse d’une théorie générale. Québec: PUL.

[ref66] Luhmann N. (2012). La réalité des médias de masse. Paris: Diaphanes.

[ref67] Luhmann N. (2021). La société de la société. Paris: Exils.

[ref68] Lunardi P. Mansk L. M. Z. Jaimes L. F. Pereira G. S. (2021). On the novel mechanisms for social memory and the emerging role of neurogenesis. Brain Res. Bull. 171, 56–66. doi: 10.1016/j.brainresbull.2021.03.006, PMID: 33753208

[ref69] Lund A. E. Russell C. (2022). What is the relationship between collective memory and metacognition? Prog. Brain Res. 274, 31–70. doi: 10.1016/bs.pbr.2022.07.006, PMID: 36167451

[ref70] Luo R. Feng W. Xu Y. (2022). Collective memory and social imaginaries of the epidemic situation in COVID-19-based on the qualitative research of college students in Wuhan China. Front Psychol. 13:998121. doi: 10.3389/fpsyg.2022.1058944, PMID: 36211935 PMC9539215

[ref71] Mary A. Dayan J. Leone G. Postel C. Fraisse F. Malle C.. (2020). Resilience after trauma: the role of memory suppression. Science 367:6479. doi: 10.1126/science.aay8477 32054733

[ref9012] Maswood R. Rasmussen A. S. Rajaram S. (2019). Collaborative remembering of emotional autobiographical memories: implications for emotion regulation and collective memory. J. Exp. Psychol. Gen. 148, 65–79. doi: 10.1037/xge0000468, PMID: 30211580

[ref72] Maturana H. R. Varela F. (1994). L’Arbre de la connaissance. Racines biologiques de la compréhension humaine. Paris: Addison-Wesley France.

[ref9103] Mead G. H. (2006). L’esprit, le soi et la société. Paris: PUF.

[ref9013] Meier B. (2021). Collective memory for political leaders in a collaborative government system: evidence for generation-specific reminiscence effects. Mem. Cognit. 49, 83–89. doi: 10.3758/s13421-020-01076-8, PMID: 32761310 PMC7819937

[ref9014] Mendes L. O. Cunha L. R. Mendes R. S. (2022). Popularity of video games and collective memory. Entropy (Basel). 24:860. doi: 10.3390/e24070860, PMID: 35885084 PMC9320117

[ref9015] Merck C. Yamashiro J. K. Hirst W. (2020). Remembering the big game: social identity and memory for media events. Memory 28, 795–814. doi: 10.1080/09658211.2020.1784232, PMID: 32588742

[ref73] Misztal B. A. (2003). Durkheim on collective memory. J. Class. Sociol. 3, 123–143. doi: 10.1177/1468795X030032002

[ref74] Momennejad I. Duker A. Coman A. (2019). Bridge ties bind collective memories. Nat. Commun. 10:1578. doi: 10.1038/s41467-019-09452-y, PMID: 30952861 PMC6451000

[ref801] Muller F. Bermejo F. Hirst W. (2016). Argentines’ collective memories of the military Junta of 1976: differences and similarities across generations and ideology. Memory 24, 990–1006. doi: 10.1080/09658211.2015.1061013 26293779

[ref802] Mutlutürk A. Tekcan A. I. Boduroglu A. (2022). Stability and change in the organisation of collective memory representations. Memory 30, 1302–1318. doi: 10.1080/09658211.2022.2112232, PMID: 35974671

[ref803] Nelson K. (2003). Self and social functions: individual autobiographical memory and collective narrative. Memory 11, 125–136. doi: 10.1080/741938203 12820826

[ref75] Okuyama T. (2018). Social memory engram in the hippocampus. Neurosci. Res. 129, 17–23. doi: 10.1016/j.neures.2017.05.007, PMID: 28577978

[ref76] Olick J. K. (1999). Collective memory: the two cultures. Sociol Theory 17, 333–348. doi: 10.1111/0735-2751.00083, PMID: 37651345

[ref77] Olick J. K. (2009). Between chaos and diversity: is social memory studies a field? Int. J. Polit. Cult. Soc. 22, 249–252.

[ref78] Olick J. K. Levy D. (1997). Collective memory and cultural constraint: holocaust myth and rationality in German politics. Am. Sociol. Rev. 62, 921–936. doi: 10.2307/2657347

[ref79] Olick J. K. Robbins J. (1998). Social memory studies: from “collective memory” to the historical sociology of mnemonic practices. Annu. Rev. Sociol. 24, 105–140. doi: 10.1146/annurev.soc.24.1.105

[ref80] Orianne J.-F. (2023). Mémoire collective et mémoire sociale: apports de la sociologie à une théorie générale de la mémoire. Biol Aujourdhui 217, 65–72. doi: 10.1051/jbio/2023003, PMID: 37409866

[ref81] Parsons T. (1937). The structure of social action. New York: MacGraw Hill.

[ref82] Parsons T. (1951). The social system. New York: The FreePress.

[ref83] Parsons T. (1963). Éléments pour une sociologie de l’action. Paris: Plon.

[ref84] Parsons T. (2004). La configuration du système social. Toulouse: Presses de l’Université des sciences sociales de Toulouse.

[ref85] Rajaram S. (2022). Collective memory and the individual mind. Trends Cogn. Sci. 26, 1056–1058. doi: 10.1016/j.tics.2022.09.014, PMID: 36272938

[ref86] Rajaram S. Pereira-Pasarin L. P. (2010). Collaborative memory: cognitive research and theory. Perspect. Psychol. Sci. 5, 649–663. doi: 10.1177/1745691610388763, PMID: 26161882

[ref87] Reese E. Fivush R. (2008). The development of collective remembering. Memory 16, 201–212. doi: 10.1080/09658210701806516, PMID: 18324547

[ref804] Roediger H. L. 3rd. (2021). Three facets of collective memory. Am. Psychol. 76, 1388–1400. doi: 10.1037/amp0000938, PMID: 35266734

[ref805] Roediger H. L. 3rd. Abel M. (2015). Collective memory: a new arena of cognitive study. Trends Cogn. Sci. 19, 359–361. doi: 10.1016/j.tics.2015.04.003, PMID: 25953047

[ref88] Roediger H. L. Meade M. L. Bergman E. T. (2001). Social contagion of memory. Psychon. Bull. Rev. 8, 365–371. doi: 10.3758/BF03196174, PMID: 11495127

[ref9104] Russell N. (2006). Collective Memory before and after Halbwachs. The Fr. Rev. 79, 792–804.

[ref9105] Sabourin P. (1997). Perspective sur la mémoire sociale de Maurice Halbwachs. Sociologie et sociétés. 29, 139–161.

[ref89] Savelsberg J. J. King R. D. (2007). Law and collective memory. Annu. Rev. Law Soc. Sci. 3, 189–211. doi: 10.1146/annurev.lawsocsci.3.081806.112757, PMID: 10407743

[ref90] Saxena K. Morris R. G. (2016). Social memory goes viral. Science 353, 1496–1497. doi: 10.1126/science.aai7788, PMID: 27708089

[ref91] Schwartz B. (1982). The social context of commemoration: a study in collective memory. Soc. Forces 61, 374–402. doi: 10.2307/2578232, PMID: 32082507

[ref92] Schwartz B. (1991). Social change and collective memory: the democratization of George Washington. Am. Sociol. Rev. 56, 221–236. doi: 10.2307/2095781

[ref93] Schwartz B. (1997). Collective memory and history: how Abraham Lincoln became a symbol of racial equality. Sociol. Q. 38, 469–496. doi: 10.1111/j.1533-8525.1997.tb00488.x

[ref94] Searle J. (1995). La construction de la réalité sociale. Paris: Gallimard.

[ref95] Smith A. L. (2004). Heteroglossia, “common sense,” and social memory. Am. Ethnol. 31, 251–269. doi: 10.1525/ae.2004.31.2.251

[ref96] Smith A. S. Williams Avram S. K. Cymerblit-Sabba A. Song J. Young W. S. (2016). Targeted activation of the hippocampal CA2 area strongly enhances social memory. Mol. Psychiatry 21, 1137–1144. doi: 10.1038/mp.2015.189, PMID: 26728562 PMC4935650

[ref97] Spencer-Brown G. (1969). Laws of form. London: Allen & Unwin.

[ref806] Spivack S. Philibotte S. J. Spilka N. H. Passman I. J. Wallisch P. (2019). Who remembers the Beatles? The collective memory for popular music. PLoS One 14:e0210066. doi: 10.1371/journal.pone.0210066, PMID: 30726220 PMC6364888

[ref9106] Strauss A. (1992). Miroirs et masques. Paris: Métailié.

[ref98] Sutton J. (2008). Between individual and collective memory: coordination, interaction, distribution. Soc. Res. 75, 23–48. doi: 10.1353/sor.2008.0063, PMID: 37066599

[ref99] Teubner G. (1996), Droit et réflexivité. Paris: LGDJ.

[ref807] Tileagă C. (2012). Communism and the meaning of social memory: towards a critical-interpretive approach. Integr. Psychol. Behav Sci. 46, 475–492. doi: 10.1007/s12124-012-9207-x, PMID: 22718124

[ref100] Thierry B. Theraulaz G. Gautier J. Y. Stiegler B. (1995). Joint memory. Behav. Process. 35, 127–140. doi: 10.1016/0376-6357(95)00039-9, PMID: 24896025

[ref101] Tse D. Langston R. F. Kakeyama M. Bethus I. Spooner P. A. Wood E. R.. (2007). Schemas and memory consolidation. Science 316, 76–82. doi: 10.1126/science.1135935, PMID: 17412951

[ref102] Tulving E. (1985). How many memory systems are there? Am. Psychol. 40, 385–398. doi: 10.1037/0003-066X.40.4.385, PMID: 37742186

[ref103] van der Kooij M. A. Sandi C. (2012). Social memories in rodents: methods, mechanisms and modulation by stress. Neurosci. Biobehav. Rev. 36, 1763–1772. doi: 10.1016/j.neubiorev.2011.10.006, PMID: 22079398

[ref104] Van Swol L. M. (2008). Performance and process in collective and individual memory: the role of social decision schemes and memory bias in collective memory. Memory 16, 274–287. doi: 10.1080/09658210701810187, PMID: 18324552

[ref105] Varela F. (2017). Le cercle créateur. Paris: Seuil.

[ref106] Vincent G. E. (1916). The social memory. Minn. Hist. Bull. 1, 249–259.

[ref107] Vinitzky-Seroussi V. Teeger C. (2010). Unpacking the unspoken: silence in collective memory and forgetting. Soc. Forces 88, 1103–1122. doi: 10.1353/sof.0.0290

[ref701] Wang Q. (2008). On the cultural constitution of collective memory. Memory 16, 305–317. doi: 10.1080/09658210701801467, PMID: 18324554

[ref702] Wang Q. (2021). The cultural foundation of human memory. Annu Rev. Psychol. 72, 151–179. doi: 10.1146/annurev-psych-070920-023638, PMID: 32928062

[ref703] Weldon M. S. Bellinger K. D. (1997). Collective memory: collaborative and individual processes in remembering. J. Exp. Psychol. Learn Mem. Cogn. 23, 1160–1175. doi: 10.1037/0278-7393.23.5.1160, PMID: 9293627

[ref108] Wertsch J. V. (2008a). The narrative Organization of Collective Memory. Ethos 36, 120–135. doi: 10.1111/j.1548-1352.2008.00007.x

[ref109] Wertsch J. V. (2008b). Collective memory and narrative templates. Soc. Res. 75, 133–156. doi: 10.1353/sor.2008.0051

[ref110] Wertsch J. V. Jäggi O. L. (2022). Habits of collective memory. Prog. Brain Res. 274, 149–166. doi: 10.1016/bs.pbr.2022.07.005, PMID: 36167447

[ref704] Wertsch J. V. Roediger H. L. (2008). Collective memory: conceptual foundations and theoretical approaches. Memory 16, 318–326. doi: 10.1080/09658210701801434, PMID: 18324555

[ref112] Wilson R. A. (2005). Collective memory, group minds, and the extended mind thesis. Cogn. Process. 6, 227–236. doi: 10.1007/s10339-005-0012-z, PMID: 18239951

[ref601] Yamashiro J. K. Hirst W. (2020). Convergence on collective memories: central speakers and distributed remembering. J. Exp. Psychol. Gen. 149, 461–481. doi: 10.1037/xge0000656, PMID: 31318259

[ref602] Yamashiro J. K. Roediger H. L. 3rd. (2021). Biased collective memories and historical overclaiming: an availability heuristic account. Mem. Cognit. 49, 311–322. doi: 10.3758/s13421-020-01090-w, PMID: 32844381

[ref603] Yasseri T. Gildersleve P. David L. (2022). Collective memory in the digital age. Prog Brain Res. 274, 203–226. doi: 10.1016/bs.pbr.2022.07.001, PMID: 36167450

[ref604] Zaromb F. Butler A. C. Agarwal P. K. Roediger H. L. 3rd. (2014). Collective memories of three wars in United States history in younger and older adults. Mem. Cognit. 42, 383–399. doi: 10.3758/s13421-013-0369-7, PMID: 24097190

